# The *in silico* and *in vitro* analysis of donepezil derivatives for *Anopheles* acetylcholinesterase inhibition

**DOI:** 10.1371/journal.pone.0277363

**Published:** 2022-11-09

**Authors:** Thankhoe A. Rants’o, Divan G. van Greunen, C. Johan van der Westhuizen, Darren L. Riley, Jenny-Lee Panayides, Lizette L. Koekemoer, Robyn L. van Zyl

**Affiliations:** 1 Pharmacology Division, Department of Pharmacy and Pharmacology, Faculty of Health Sciences, University of the Witwatersrand, Johannesburg, South Africa; 2 WITS Research Institute for Malaria (WRIM), Faculty of Health Sciences, University of the Witwatersrand, Johannesburg, South Africa; 3 Department of Chemistry, Natural and Agricultural Sciences, University of Pretoria, Tshwane, South Africa; 4 Pharmaceutical Technologies, CSIR Future Production: Chemicals, Tshwane, South Africa; 5 School of Pathology, Faculty of Health Sciences, University of the Witwatersrand, Johannesburg, South Africa; 6 Centre for Emerging Zoonotic and Parasitic Diseases, National Institute for Communicable Diseases of the National Health Laboratory Service, Johannesburg, South Africa; Al-Azhar University, EGYPT

## Abstract

Current studies on *Anopheles* anticholinesterase insecticides are focusing on identifying agents with high selectivity towards *Anopheles* over mammalian targets. Acetylcholinesterase (AChE) from electric eel is often used as the bioequivalent enzyme to study ligands designed for activity and inhibition in human. In this study, previously identified derivatives of a potent AChE, donepezil, that have exhibited low activity on electric eel AChE were assessed for potential AChE-based larvicidal effects on four African malaria vectors; *An*. *funestus*, *An*. *arabiensis*, *An*. *gambiae* and *An*. *coluzzii*. This led to the identification of four larvicidal agents with a lead molecule, 1-benzyl-*N*-(thiazol-2-yl) piperidine-4-carboxamide **2** showing selectivity for *An*. *arabiensis* as a larvicidal AChE agent. Differential activities of this molecule on *An*. *arabiensis* and electric eel AChE targets were studied through molecular modelling. Homology modelling was used to generate a three-dimensional structure of the *An*. *arabiensis* AChE for this binding assay. The conformation of this molecule and corresponding interactions with the AChE catalytic site was markedly different between the two targets. Assessment of the differences between the AChE binding sites from electric eel, human and *Anopheles* revealed that the electric eel and human AChE proteins were very similar. In contrast, *Anopheles* AChE had a smaller cysteine residue in place of bulky phenylalanine group at the entrance to the catalytic site, and a smaller aspartic acid residue at the base of the active site gorge, in place of the bulky tyrosine residues. Results from this study suggest that this difference affects the ligand orientation and corresponding interactions at the catalytic site. The lead molecule **2** also formed more favourable interactions with *An*. *arabiensis* AChE model than other *Anopheles* AChE targets, possibly explaining the observed selectivity among other assessed *Anopheles* species. This study suggests that 1-benzyl-*N*-(thiazol-2-yl) piperidine-4-carboxamide **2** may be a lead compound for designing novel insecticides against *Anopheles* vectors with reduced toxic potential on humans.

## 1. Introduction

**Fig 1:**
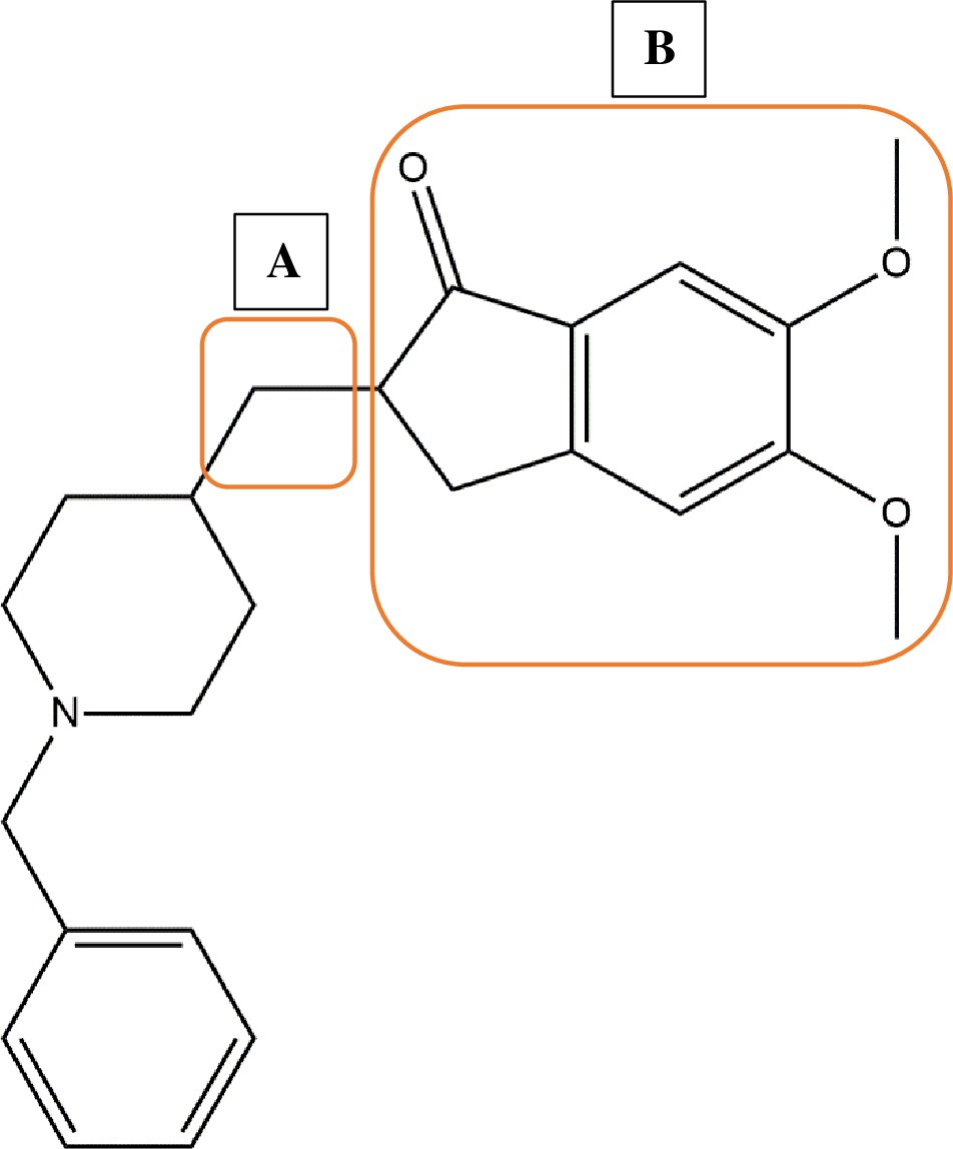
Chemical structure of donepezil showing two common sides of derivatization: A) indanone moiety and B) methyl linker.

Donepezil (1-benzyl-4-((5,6-dimethoxy-1-indanon)-2-yl)methylpiperidine) shown in Fig 1 is a known potent human acetylcholinesterase (AChE) inhibitor used clinically in the management of symptoms associated with mild to severe Alzheimer’s disease [1, 2]. The derivatisation of donepezil has been pursued to produce more active AChE agents against the human target with several studies have shown evidence of derivatives with high potency [3–7]. Particularly, van Greunen *et al*. [4] reported a potent derivative by converting the methyl linker (part A; Fig 1) between the piperidine ring and indanone group to an ester linker. This lead compound displayed a 50% inhibitory concentration (IC_50_) value (0.03 ± 0.07 μM) similar to that of donepezil (0.05 ± 0.06 μM) when screened *in vitro* against AChE from *Electrophorus electricus* (electric eel) [4]. The AChEs from the electric eel and human have been shown to display similar activities, kinetics and inhibition profiles, as a result, electric eel AChE is used as a less expensive alternative to human AChE in bioassays [8, 9]. In a follow-up study, van Greunen and colleagues [3] synthesized and assessed various analogues of this lead compound for improved AChE activity. These new analogues featured the substitution of the ester linker found between the indanone and piperidine ring systems in their previous hit, by an amide that is more stable against rapid metabolism. In addition, the indanone (part B; Fig 1) was replaced with various aryl and aromatic groups [10]. Though at least two analogues were considerably active (IC_50_ <10 μM), none were as active as donepezil against electric eel AChE, and some were biologically inactive (IC_50_ >100 μM) [3].

Current studies on *Anopheles* AChEs are aimed at achieving high selectivity towards *Anopheles* over mammalian targets to reduce human toxicity [11–14]. Utilizing the molecular differences between human and insect AChE binding sites, these studies target conserved amino acid residues in *Anopheles* and compounds with selectivity index more than 100-fold towards *Anopheles* AChE have thus far been reported [11, 12]. The current study assessed the donepezil derivatives prepared by van Greunen *et al*. [3] for potential *Anopheles* AChE inhibition and rationalised their binding profiles through molecular docking. Interestingly, the parent drug, donepezil, has been proven to be active against insect AChE [15, 16], but is known to be approximately 40 times more selective to human AChE than the corresponding *Anopheles* target [16].

## 2. Materials and methods

**Fig 2:**
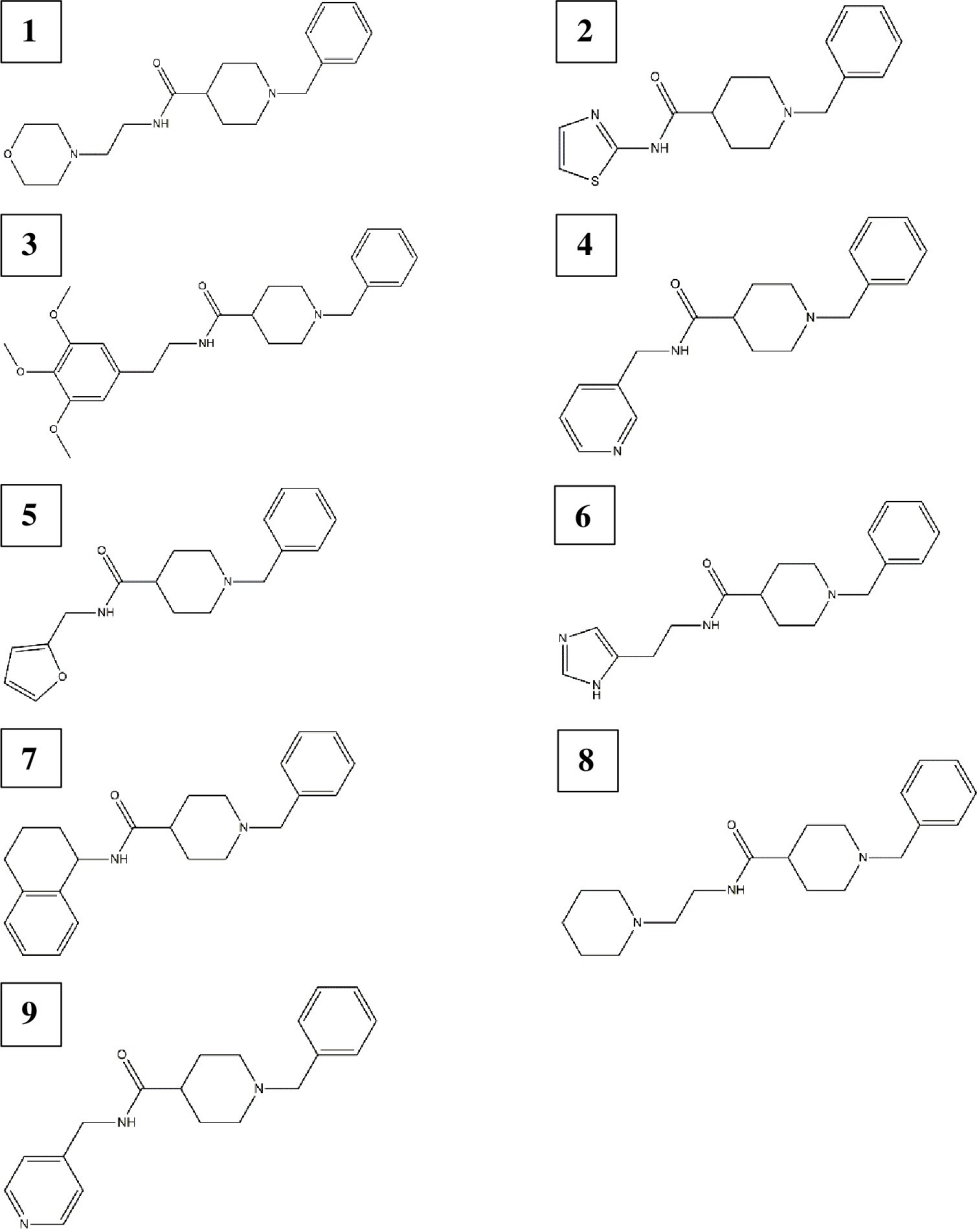
Chemical structures of the nine donepezil derivatives screened. 1-benzyl-*N*-(2-morpholinoethyl) piperidine-4-carboxamide **1**, 1-benzyl-*N*-(thiazol-2-yl) piperidine-4-carboxamide **2**, 1-benzyl-*N*-(3,4,5-trimethoxyphenethyl) piperidine-4-carboxamide **3**, 1-benzyl-*N*-(pyridine-3-ylmethyl) piperidine-4-carboxamide **4**, 1-benzyl-*N*-(furan-2-ylmethyl) piperidine-4-carboxamide **5**, *N*-[2-(1H-imidazol-4-yl)ethyl]-1-benzylpiperidine-4- carboxamide **6**, 1-benzyl-*N*-(1,2,3,4-tetrahydro-naphthalen-1-yl) piperidine-4-carboxamide **7**, 1-benzyl-*N*-(2-(piperidin-1-yl)ethyl)piperidine-4-carboxamide **8**, and 1-benzyl-*N*-(pyridine-4-ylmethyl) piperidine-4-carboxamide **9** [3].

This study received the animal research ethics waiver (Waiver Number: 07-11-2017-O) from Wits Animal Research Ethics Committee. Nine donepezil derivatives (Fig 2) with low activity (LC_50_ values >50 μM) against electric eel AChE from a previous study [3] were used for this research. Acetylthiocholine iodide, 5,5′-dithiobis(2-nitrobenzoic acid) (DTNB), dimethylsulfoxide (DMSO), Triton X-100, potassium dichromate, propoxur and sodium phosphate buffer (dibasic) (Na_2_HPO_4_) were bought from Sigma Aldrich (South Africa) with a purity >90%_._

### 2.1. *Anopheles spp*. rearing

The laboratory-reared colonies of *An*. *funestus* (FUMOZ), *An*. *arabiensis* (KWAG), *An*. *gambiae* (COGS) and *An*. *coluzzii* (G3) were used for larvicidal and AChE screening assays. These are common *Anopheles* species responsible for malaria transmission in Africa [17–20]. These colonies were maintained under standard insectary conditions as reported by Hunt *et al*. [21] and Zengenene *et al*. [22]. FUMOZ was first collected from Mozambique in 2000 and has a low-level intensity of pyrethroid and carbamate resistance [23–25]. KWAG was collected from KwaZulu-Natal (South Africa) in 2005 and has shown resistance to pyrethroids and the organochloride, dichloro-diphenyl-trichloroethane (DDT) [19, 26]. On the other hand, COGS was collected in 2009 from Congo and displays resistance to multiple insecticides such as pyrethroids, organochlorides and carbamates [27, 28].

### 2.2. Larvicidal assay

The larval toxicity of novel donepezil analogues was assessed using the World Health Organization (WHO) bioassay for testing mosquito larvicides [29]. Briefly, batches of 20 third-instar larvae of each colony were transferred into the test cups into which 250 μL of a specific donepezil derivative was added. The incubation mixture was performed in a total volume of 250 mL of deionized water under 27°C and humidity ≥78%. The test compounds were dissolved in DMSO and assessed for larvicidal activity at 10 times increasing concentrations from 0.0005 μM to 500 μM. Propoxur, a standard larvicide and an AChE agent [30, 31], was used as a positive control, while DMSO was employed for the negative vehicle control. Larval mortality was recorded in 24, 48 and 72 hours and larvae were fed with protein dog food at day 0 and after every mortality counting [32].

### 2.3. Brine shrimp lethality assay

Artificial seawater was prepared by dissolving 32 g of Tropic Marine^®^ Sea salt in 1L of deionized water. The seawater was poured into an inverted plastic bottle after which the brine shrimp eggs were added for hatching. Regular airflow was supplied to the seawater to continually disperse the eggs and oxygenate the water. Moreover, a concentrated light was supplied from a lamp (220–240 V, 15W) to provide warmth to optimize hatching conditions for the 24 h incubation time [33]. Following this, the cytotoxicity potential of the donepezil derivatives was evaluated by the brine shrimp lethality assay using *Artemia franciscana* [34]. The same concentrations used in the larvicidal assessment were also used for the toxicity evaluation. Inside 48-well plates, 50 μL of the test compound was incubated with 30–50 nauplii in 450 μL of the seawater. The wells were then observed under the stereo microscope (Olympus) at 10X magnification for dead nauplii and the induced mortality was recorded after 24 h. Where mortality was observed, the morphological changes were observed at 10X magnification using the stereo microscope mounted with a Dino-Eye camera. For the negative control, DMSO was used in place of the test compound, while potassium dichromate was used as a positive control [35, 36].

### 2.4. AChE assay

The evaluation of AChE activity of the donepezil derivatives was conducted using the modified Ellman assay [11, 37]. Mosquitoes from the four *Anopheles* colonies were separately homogenized in Na_2_HPO_4_, 1% Triton X-100 (pH 8.0) and used as an enzyme source. Protein content was assessed using the standard Lowry protein assay [38]. The incubation mixture in a 96-well plate consisted of 20 μl of the enzyme in 132 μL of the assay buffer (Na_2_HPO_4_, 1% Triton X-100; pH 8.0). Twenty (20) μL of the donepezil derivatives at concentrations ranging from 0.0005 μM to 500 μM dissolved in DMSO were added. Ellman’s reagent, 5,5′-dithiobis(2-nitrobenzoic acid) (DTNB), was freshly prepared in sodium phosphate buffer (dibasic; pH 7.0) and 8 μL (0.2 mM) added to the incubation mixture. To initiate the reaction, 20 μL (1 mM) of the AChE substrate, acetylthiocholine iodide was added making a total volume of 200 μL in each well and the absorbance readings were obtained using the UV-Visible spectrophotometer at 412 nm. Propoxur was kept as a positive control on the basis of having both AChE and larvicidal activities [31], while DMSO was used as a negative control. DMSO is known to exhibit AChE inhibition at concentrations above 1% [39], as such the highest DMSO concentration used in this study was 0.4%. For screening against electric eel AChE, donepezil was used as a positive control due to its known potent inhibitory activity against this target [3, 4]. However, the insecticide propoxur was also assessed against this target to determine AChE selectivity between electric eel and *Anopheles* and subsequent comparison with the test compounds.

### 2.5. *In silico* studies

#### 2.5.1. Homology modelling

As a crystal structure of *An*. *arabiensis* was not available, it was elected to employ the use of homology modelling [40]. This approach involved four successive steps: (i) target amino acid sequence identification, (ii) template identification, (iii) sequence alignment between target and template, and (iv) model building and optimization [41, 42]. Homology modelling is considered the most accurate *in silico* approach to generate 3D models of proteins [42], however, certain minimum requirements had to be met. To produce a reliable protein structure, an existing sequence that matched at least 30% of the target sequence should be used as a template. These are known to display similar structures and interaction mechanisms [43–45]. Moreover, for the 30% identity cut-off, the length of the aligned sequences between the two should be >100 amino acid residues [46]. Sequence identity without taking into account the length of aligned amino acid residues, has been shown to result in less accurate models [47].

The amino acid sequence of *An*. *arabiensis* was retrieved from UniProt Knowledge Base (Accession number: A0A182HKN4). This accession number was submitted to the SWISS-MODEL database server for a reference 3D structure search and model building [48]. *An*. *gambiae* AChE (PDB: 5YDI) was selected as a template [49]. UCSF Chimera v1.16 was used for model optimization and visualization [50, 51]. Moreover, the correctness of the model was checked through ProQ webserver [52], and the model was validated with Verify3D and MolProbity [53, 54].

#### 2.5.2. Molecular docking

Schrödinger Release 2018–2 molecular docking package, Maestro version 12.9, (Schrödinger LLC, New York) was used for ligand binding assessments [55]. The generated *An*. *arabiensis* AChE 3D structure and PDB sourced related proteins for comparative studies were prepared through Maestro’s protein preparation function. This included the AChE proteins from *An*. *gambiae* (PDB: 5YDI) [49]), electric eel (PDB: 1EVE [56]) and human (PDB: 4EY7 [57]). The preparation included optimization of H-bonds and removal of non-hetero groups and non-essential water molecules before minimization by OPLS3e force field [58]. The receptor grid was also generated using the OPLS3e force field to define the binding site [55]. Similarly, the ligands were prepared for docking using the LigPrep tool in which they were allowed to generate possible stereoisomers as well as ionization and tautomeric states at pH 7.0 (±2.0) [59, 60]. Finally, the extra precision mode was used for assessing the binding profiles of the prepared ligands to the receptor sites [61, 62].

### 2.6. Statistical analyses

All *in vitro* and *in vivo* assays were performed in triplicate. The 50% lethal concentration (LC_50_) values were calculated from the probit analysis method using the SPSS Statistics v28 package (International Business Machines Corporation, NY, USA) and the IC_50_ values were determined by non-linear regression analysis using GraphPad Prism 9 (GraphPad Software, CA, USA) [30, 36].

## 3. Results and discussion

### 3.1. Larvicidal activity

Only derivatives **1**, **2**, **5** and **8** (Fig 2) showed larvicidal activities (Table 1). Specifically, derivatives **1**, **5** and **8** were active against *An*. *funestus* with lower LC_50_ values (2.65, 2.96 and 0.80 μM) compared to the positive control, propoxur (9.90 μM) (Table 1). On the other hand, derivative **2** was selective to *An*. *arabiensis*. This derivative was about 10-fold (LC_50_: 0.88 μM) more potent than propoxur (LC_50_: 8.77 μM) for *An*. *arabiensis* larval toxicity (Table 1). As a result, only derivatives **1**, **2**, **5** and **8** were selected for further analysis in the AChE inhibition assay.

**Table 1:** Larvicidal activities of the assessed compounds.

	*Anopheles* colonies Larvicidal LC_50_ (95% confidence interval range) (μM) [Chi square (X^2^); degree of freedom (df); *p* value; intercept; standard error (SE)]			
Donepezil derivative	*An*. *Funestus*	*An*. *arabiensis*	*An*. *gambiae*	*An*. *coluzzii*
**1**	2.65 (1.72–4.12) [X^2^: 4.76; df: 5; *p* = 0.45^a^; intercept: -0.27; SE: 0.06]	> 100	> 100	> 100
**2**	> 100	0.88 (0.35–2.27) [X^2^: 17.56; df: 5; *p* = 0.004^b^; intercept: 0.05; SE: 0.07]	> 100	> 100
**3**	> 100	> 100	> 100	> 100
**4**	> 100	> 100	> 100	> 100
**5**	2.96 (1.94–4.57) [X^2^: 5.43; df: 5; *p* = 0.37^a^; intercept: -0.31; SE: 0.06]	> 100	> 100	> 100
**6**	> 100	> 100	> 100	> 100
**7**	> 100	> 100	> 100	> 100
**8**	0.80 (0.56–1.16) [X^2^: 3.58; df: 5; *p* = 0.61^a^; intercept: -0.78; SE: 0.07]	> 100	> 100	> 100
**9**	> 100	> 100	> 100	> 100
**Propoxur**	9.90 (2.95–41.41) [X^2^: 26.24; df: 5; *p* <0.001^b^; intercept: -0.78; SE: 0.08]	8.77 (2.81–32.71) [X^2^: 23.95; df: 5; *p* <0.001^b^; intercept: -0.75; SE: 0.08]	63.99 (38.23–116.29) [X^2^: 3.96; df: 5; *p* = 0.56^a^; intercept: -1.11; SE: 0.09]	62.68 (37.72–112.78) [X^2^: 3.58; df: 5; *p* = 0.61^a^; intercept: -1.12; SE: 0.09]

^a^ Since the significance level was greater than 0.15, a heterogeneity factor was not used in the calculation of confidence interval limits.

^b^ Since the significance level was less than 0.15, a heterogeneity factor was used in the calculation of confidence interval limits.

### 3.2. Brine shrimp lethality

None of the derivatives induced artemicidal effects (100% viability at 500 μM). This suggests relative safety of these novel compounds compared to the positive control, potassium dichromate that attained the LC_50_ value of 0.004 (0.002–0.007) μM (X^2^: 8.10; df: 5; *p* = 0.151; intercept: 1.30; SE: 0.09) in agreement with a previous assessment [36]. This was a favourable outcome for the donepezil derivatives **1**, **2**, **5** and **8** which had potent larvicidal effects (Table 1), as it suggests that when used as larvicides, these derivatives would potentially be nontoxic to other aquatic lives.

### 3.3. AChE inhibition

Though the derivatives **1**, **5** and **8** showed potent larvicidal activity against *An*. *funestus*, none of these derivatives displayed AChE activities against this colony (Table 2). This suggests that these derivatives exert larvicidal activity through a different mechanism other than AChE inhibition. Surprisingly, derivatives **1**, **5**, **8** and the positive control, propoxur, showed moderate to low activity against the *in vitro An*. *gambiae* AChE target. This was in discordance to the *in vivo* larvicidal data where these molecules were inactive. It is common to have discrepancies between *in vitro* and *in vivo* data [63–65] and it has also been shown to occur with insecticide-resistant *Anopheles* larvae [66]. In fact, *An*. *gambiae* larvae have been shown to possess pharmacokinetic barriers such as thickened cuticle that play a role in preventing compound penetration at effective concentrations [66, 67]. Interestingly, derivative **2** showed potent AChE activity specifically against *An*. *arabiensis* (IC_50_ = 6.05 ± 2.21 μM; Table 2 (log-dose response curve shown in S1 Fig)), for which it also displayed larvicidal effects (Table 1).

**Table 2:** Comparison of the *Anopheles* AChE inhibitory potential of derivatives 1, 2, 5 and 8.

*Anopheles* AChE inhibition IC_50_ ± SE (95% confidence interval range) (μM)				
Donepezil derivative	*An*. *funestus*	*An*. *arabiensis*	*An*. *gambiae*	*An*. *coluzzii*
**1**	> 100	> 100	48.85 ± 10.49 (25.77–71.93)	> 100
**2**	> 100	6.05 ± 2.21 (2.78–13.16)	> 100	> 100
**5**	> 100	> 100	31.42 ± 7.56 (14.78–48.06)	> 100
**8**	> 100	> 100	28.13 ± 6.89 (12.96–43.31)	> 100
**Propoxur**	0.89 ± 0.20 (0.45–1.33)	0.78 ± 0.16 (0.42–1.13)	26.08 ± 6.21 (12.42–39.74)	25.04 ± 6.14 (11.53–38.55)

Nevertheless, derivative **2** is known to have activity against electric eel AChE [3] and displayed an IC_50_ value of 55.70 ± 12.02 μM (Table 3). The calculated selectivity index (SI = IC_50_ electric eel AChE / IC_50_ *An*. *arabiensis* AChE) between the two AChE targets was 9.2. With the selectivity index less than 10, it may not be considered selective for *An*. *arabiensis* over electric eel AChE [68, 69]. However, in comparison to propoxur, which was essentially non-selective between *An*. *arabiensis* (IC_50_ = 0.78 ± 0.16 μM; Table 2) and electric eel (IC_50_ = 1.28 ± 0.35 μM; Table 3) in agreement with previous studies [13, 70], derivative **2** showed better potential for *Anopheles* selectivity. Furthermore, with good correlation between the larvicidal and AChE activity of derivative **2** consistently against *An*. *arabiensis*, presents it as a potential lead molecule for further derivatization into more potent and selective analogues. For this reason, the donepezil derivative **2**, 1-benzyl-*N*-(thiazol-2-yl) piperidine-4-carboxamide, was selected as a lead molecule and assessed further for its AChE binding profile.

**Table 3:** Comparison of the electric eel AChE inhibitory potential of derivatives 1, 2, 5 and 8.

Donepezil derivative	*Electric eel* AChE inhibition IC_50_ ± SE (95% confidence interval range) (μM)
**1**	>100
**2**	55.70 ± 12.02 (29.24–82.16)
**5**	>100
**8**	88.29 ± 19.90 (44.49–132.10)
**Propoxur**	1.28 ± 0.35 (0.25–2.03)

## 4. *In silico* studies

### 4.1. Homology modelling

**Fig 3:**
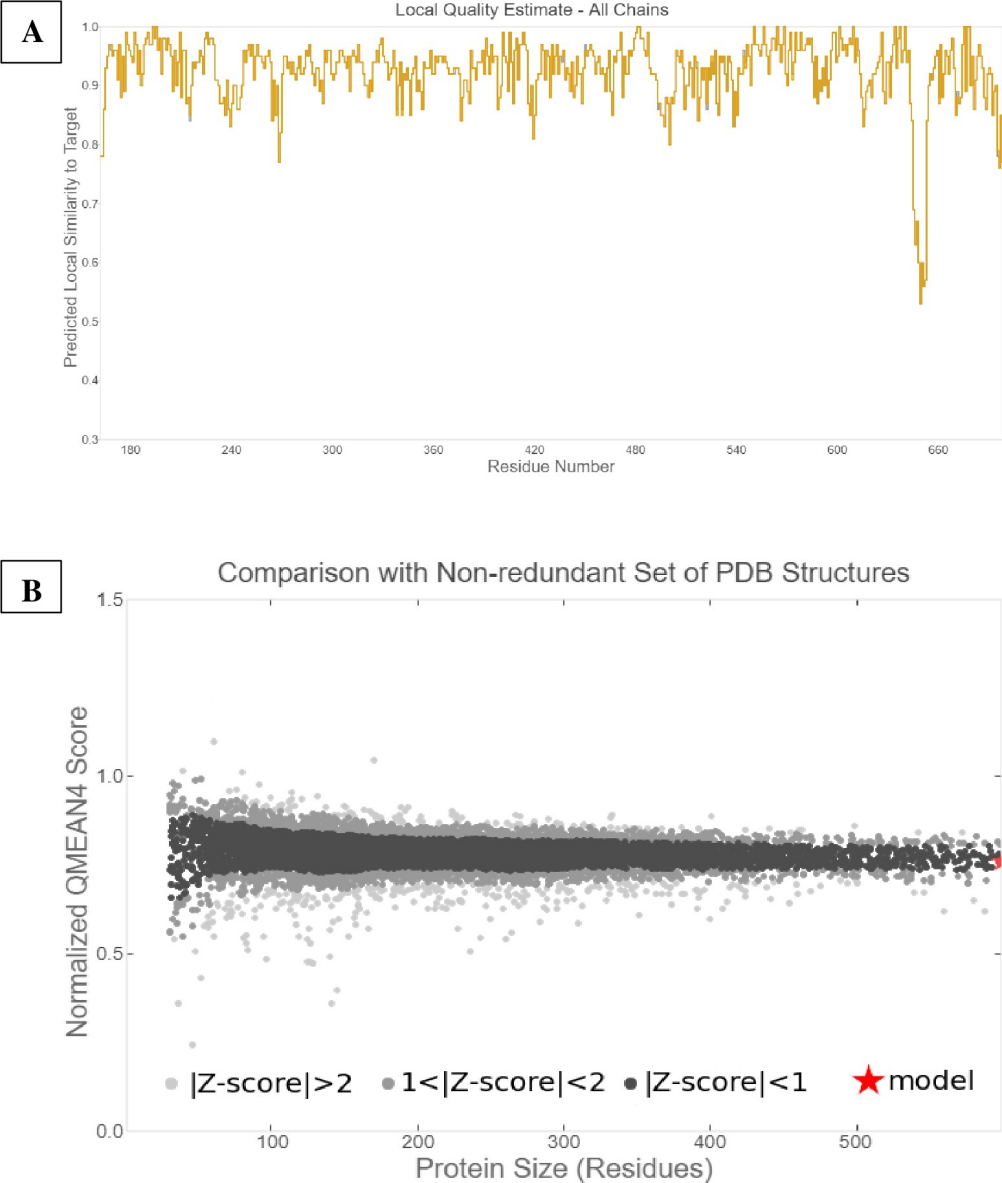
Quality of the *An*. *arabiensis* homology model. A) Shows the quality estimate of the generated model based on its similarity to the template and B) shows its comparison to non-redundant 3D structures.

The 3D AChE model for *An*. *arabiensis* was successfully built from the *An*. *gambiae* (PDB: 5YDI) template. Sequence identity between the two models was 100% with more than 500 aligned amino acid residues (range 162–698) translating into a target structural coverage of 0.73. Global model quality estimate (GMQE) and model quality estimation QMEANDisCo Global were used to estimate the overall model quality [71, 72]. The GMQE score was 0.71, while the QMEANDisCo Global achieved 0.92 ± 0.05 indicating that the final model was analogous to the experimental crystal structures. The quality of the model was assessed by comparing it to the reference and non-redundant 3D structures from PDB (Fig 3A and 3B). Similarly, quaternary structure quality estimation (QSQE) assessed the accuracy of the generated target 3D structure in terms of the inter-chain contacts in accordance with the template and resulting alignment. A value above 0.7 indicates a reliable model [48, 73] and the final model in this study reached a score of 0.74.

**Fig 4:**
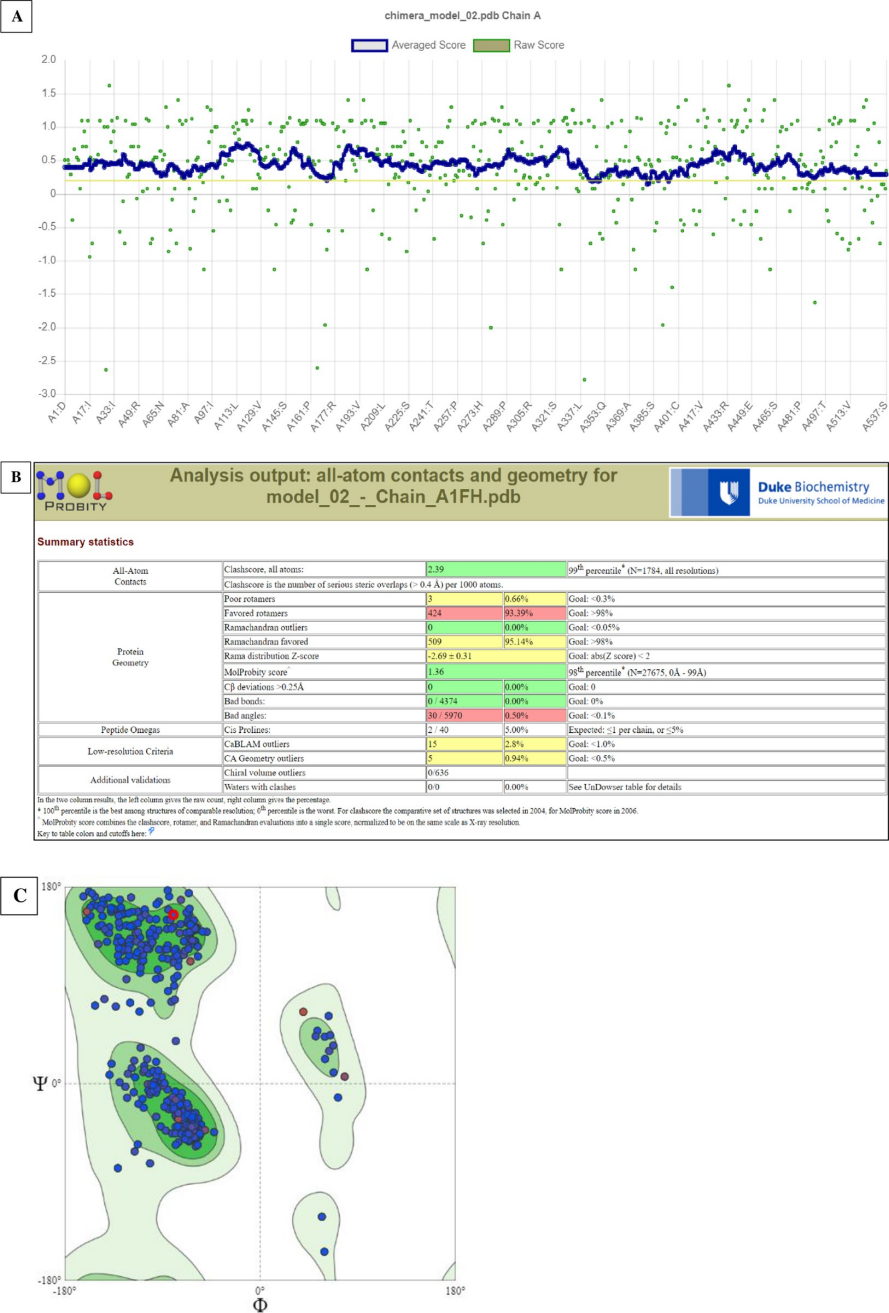
Validity of the model displayed by Verify3D tool (A) and scored by MolProbity (B) and the corresponding Ramachandran plot (C).

The assessment of the correctness of the model through ProQ showed an LGscore of 11.077. This program analysed relative frequencies of intramolecular atomic interactions within the model where the LGscore >4 indicated an extremely good model [52]. In addition, the Verify3D suggested a model PASS with 97.11% of the amino acid residues a 3D/1D score of ≥0.2 (Fig 4A). This tool assessed how compatible a generated model was with its amino acid sequences. This is assessed based on the optimum environment for each amino acid residue such as the area of the residue buried deep in the protein, and hence inaccessible to the solvent, the area of the side-chains made of polar atoms, as well as the quality of the local secondary structure [53]. A favourable MolProbity score of 1.36 and 98^th^ percentile was obtained for the model (Fig 4B) confirming the validity of the model [54]. The Ramachandran plot (Fig 4C) reported a score of 95.14% for amino acid residues in favoured regions, 99.8% in allowed regions and 0.0% for both Ramachandran outliers and Cβ deviations. This suggested a valid model with a stable backbone [74].

**Fig 5:**
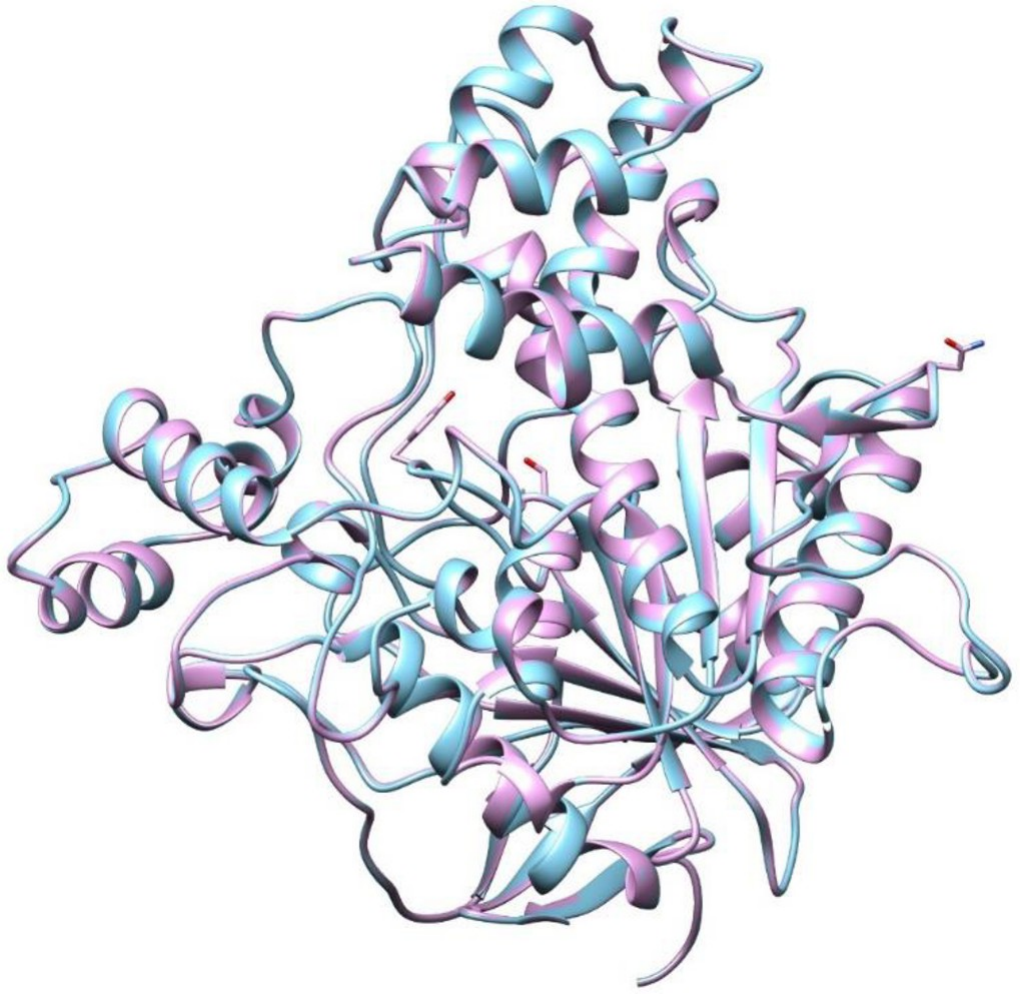
Alignment of the catalytic sites of the generated *An*. *arabiensis* AChE (sky blue) and reference *An*. *gambiae* (plum).

The new model was aligned with the existing *An*. *gambiae* AChE 3D structure (PDB: 5YDI) and visualized in UCSF Chimera. This program showed 100% alignment of the AChE catalytic sites of the modelled *An*. *arabiensis* and reference *An*. *gambiae* (Fig 5).

### 4.2. Molecular modelling

**Fig 6:**
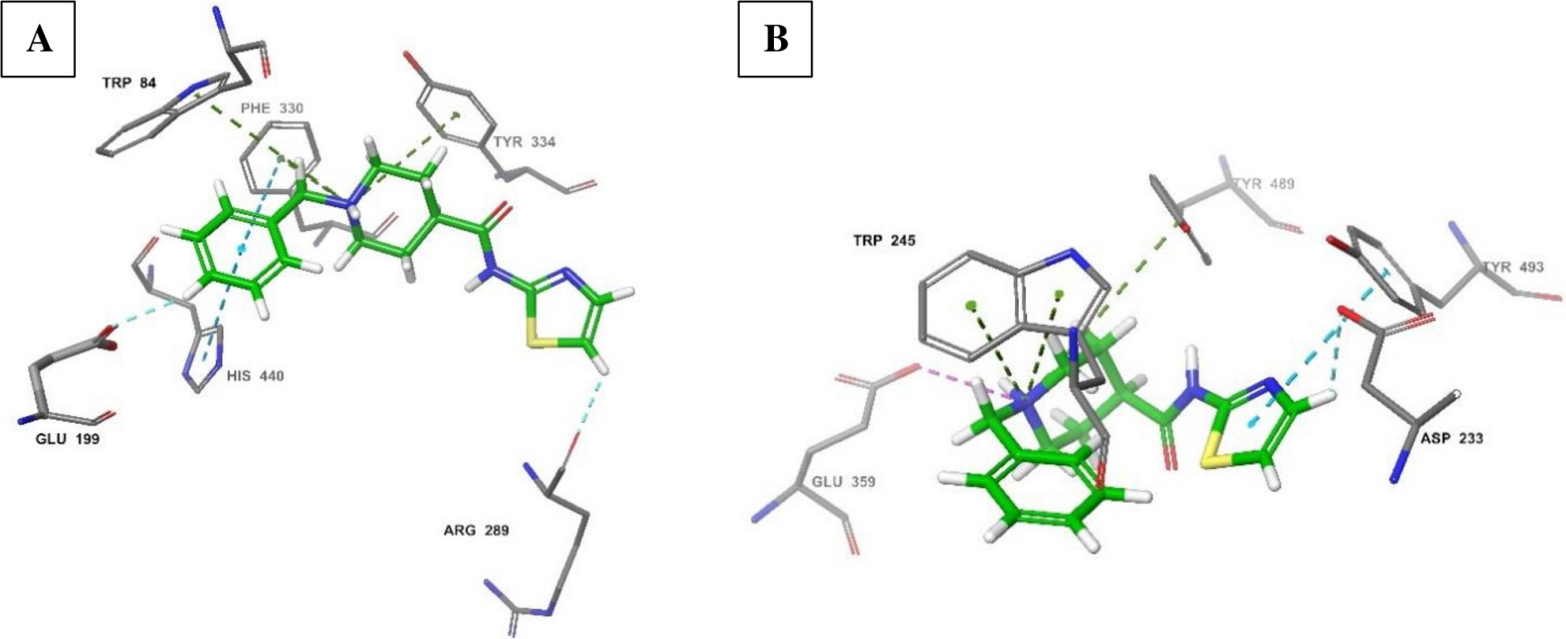
Molecular interactions of derivative **2** with electric eel AChE (A; Score: -11.8) and *An*. *arabiensis* (B; Score: -7.5).

Assessment of molecular interactions with the AChE catalytic sites of electric eel (Fig 6A) and a built model of *An*. *arabiensis* (Fig 6B) showed some similar interactions with the targets. The thiazole group was bound by arginine residues (Arg^289^ (electric eel)/Arg^233^ (*An*. *arabiensis*), however with an additional aromatic stabilization by Tyr^493^ in the *An*. *arabiensis* model (Fig 6B). Several amino acid residues were involved in the interaction with the *N*-benzylpiperidine moiety. For the electric eel, these included two catalytic triad residues Glu^199^ through hydrogen bonding and stabilization of the *N*-benzylpiperidine ring through aromatic pi-pi interaction with His^440^. This ring was held on the other side by another pi-pi interaction with Phe^330^, while Trp^84^ and Tyr^334^ formed pi-cation interactions with the ionized nitrogen of *N*-benzylpiperidine. Similarly, this nitrogen was involved in pi-cation interactions with Trp^245^ and Tyr^489^ along with Glu^359^ in the *An*. *arabiensis* model, however, with no stabilization of the *N*-benzylpiperidine ring (Fig 6B). This caused the derivative to adopt a different orientation of the *N*-benzylpiperidine group when contrasted against the conformation observed in the electric eel AChE site. This possibly caused the observed lower binding score. To gain a better understanding, the differences in the binding sites of *Anopheles*, electric eel and human AChEs from PDB were assessed.

**Fig 7:**
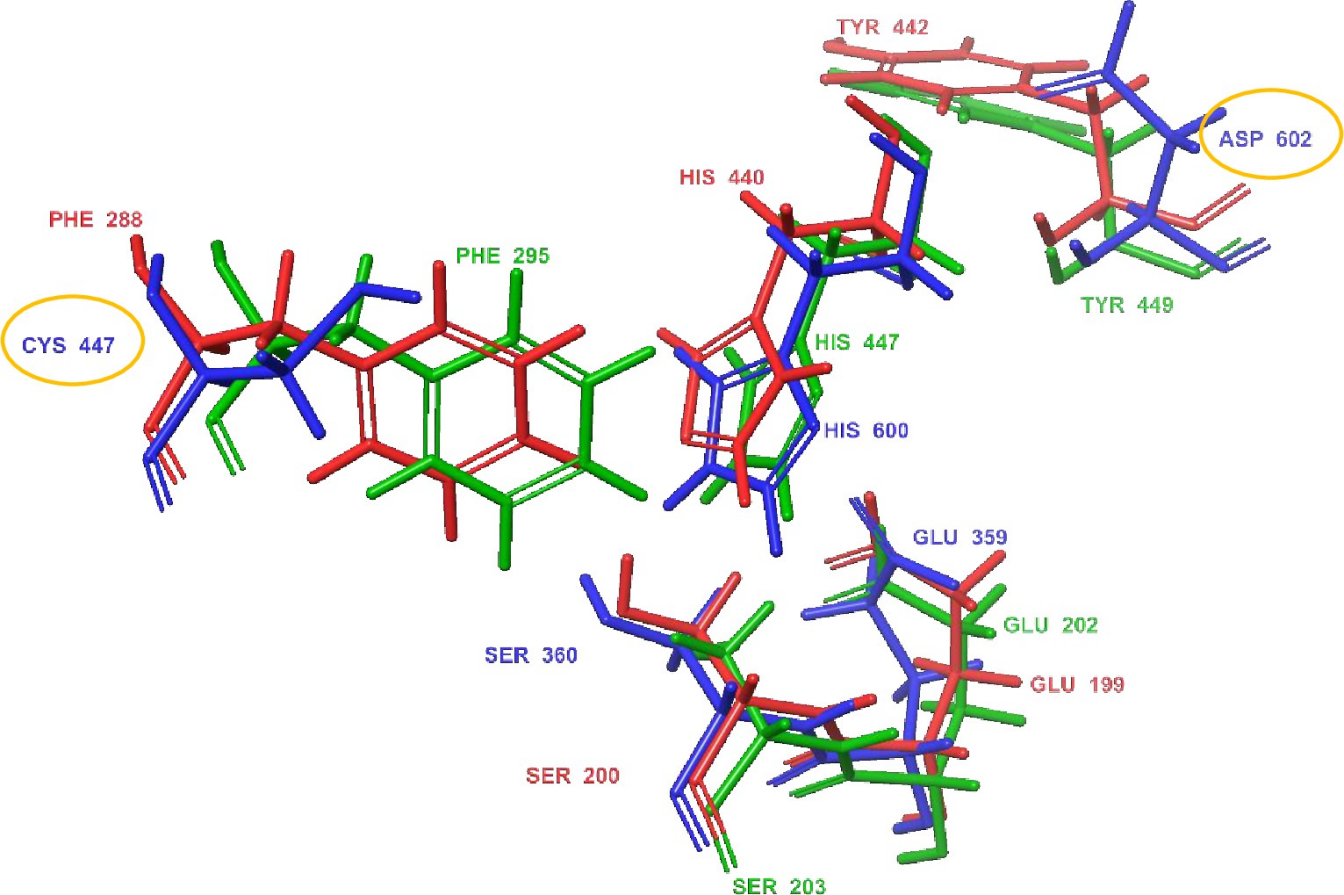
Superimposed amino acid residues showing the catalytic site entrance and the catalytic triad of electric eel (red), human (green) and *Anopheles* (blue) AChE. The distinct Cys^447^ at the *Anopheles* AChE catalytic entrance and Asp^602^ at the catalytic site base are shown by circles.

A comparison of electric eel, human and *Anopheles* AChE catalytic sites was performed by superimposing amino acid residues that represent the entrance to the catalytic site and the catalytic site amino acids through the Glide’s Quick Align function. PDB 1EVE was used for electric eel [56], PDB 4EY7 for human [57], and PDB 5YDI for the *Anopheles* (*An*. *gambiae*) AChE target [49]. The key observable difference between *Anopheles* AChE and the two other proteins was the replacement of a larger phenylalanine (Phe^288^ (human)/Phe^295^ (electric eel)) with a smaller cysteine residue (Cys^447^) at the entrance of the catalytic site. Similarly, a smaller aspartic acid residue (Asp^602^) was identified at the base of the *Anopheles* AChE catalytic site, in place of the bulky tyrosine residues (Tyr^442^ (human)/Tyr^449^ (electric eel)) (Fig 7) in agreement with the reported literature [75].

**Fig 8:**
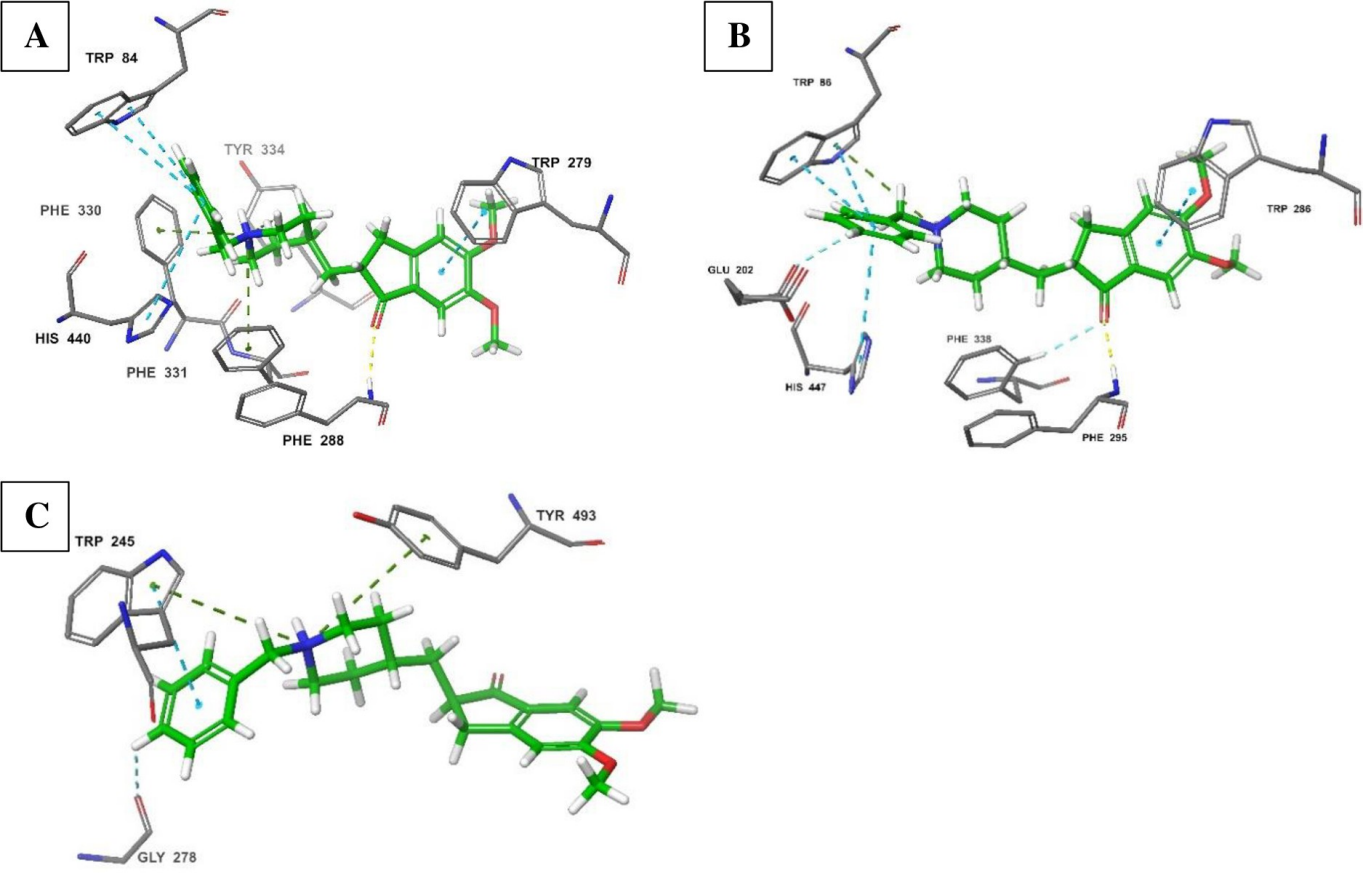
Comparison of the molecular interactions of donepezil with the AChE catalytic sites of electric eel (A; PDB: 1EVE; score: -15.0), human (B; PDB: 4EY7; score: -16.2) and *An*. *gambiae* (C; PDB: 5YDI; score: -10.2).

To gain further insights into the influence of these differences in ligand binding, the potential differences between the binding poses of the known human and electric eel AChE inhibitor, donepezil [2, 3, 5], when bound to these three AChE proteins were assessed (Fig 8). Donepezil displayed a similar binding pose and interactions with both electric eel and human AChE targets (Fig 8A and 8B). At both sites, donepezil was stabilized by aromatic interactions through the corresponding tryptophan residues. The donepezil indanone aromatic ring interacted with Trp^279^ and Trp^286^ in electric eel and human AChEs, respectively; while its *N*-benzylpiperidine ring established pi-pi stacking with Trp^84^ in electric eel and Trp^86^ in human target. Additionally, the catalytic site histidine residues (His^440^; electric eel and His^447^; human) also established the aromatic pi-pi interactions with the *N*-benzylpiperidine ring. However, in the human target, Glu^202^ was also involved in this interaction. The phenylalanine residues played a critical role in maintaining the crystal pose of donepezil through hydrogen bonding. This was achieved through Phe^288^ residue in electric eel and the comparable Phe^295^ in human AChE. Likewise, the pi-cation interactions between the nitrogen atom of *N*-benzylpiperidine and aromatic amino acids stabilized donepezil in the binding pose. In electric eel AChE, these interactions were generated from Phe^330^, Phe^331^ and Tyr^334^, while only Trp^86^ established this interaction in the corresponding human target (Fig 8A and 8B). In support of the binding similarity observed in this study, the binding potency of donepezil against electric eel and human AChE has also been reported to be similar (IC_50_ 0.035 μM and 0.030 μM, respectively) [76, 77].

On the other hand, donepezil generated different interactions with the *Anopheles* AChE from those obtained in electric eel and human targets (Fig 8C). There was no stabilization of the indanone moiety. However, the *N*-benzylpiperidine group was stabilized by Trp^245^ residues that are comparable to Trp^279^ and Trp^286^ in electric eel and human targets, respectively. The pi-cation interaction with the *N*-benzylpiperidine portion was established by the Trp^245^ and Tyr^493^ residues. This caused the aromatic ring of the *N*-benzylpiperidine to face down towards Gly^278^ residue and away from the catalytic triad amino acid histidine (His^359^; *Anopheles*) that played a key role in the binding of donepezil in electric eel (His^440^) and human (His^447^) AChEs. For the first time, this study shows that donepezil adopts a different binding pose in the *Anopheles* target, lending support to previous studies that showed significant differences in selectivity for donepezil between human and *Anopheles* AChEs [16].

Finally, as derivative **2** displayed selective inhibition of AChE for *An*. *arabiensis* over those from *An*. *gambiae*, *An*. *coluzzii* and *An*. *funestus*, the binding pose difference across the assessed *Anopheles* colonies was assessed. However, the only available crystal structures from PDB repository were from *An*. *gambiae* [49, 78]. Therefore, additional models were generated from *An*. *coluzzi* (UniProt accession number: A0A6E8V9T9) and *An*. *funestus* AChE amino acid sequences (UniProt accession number: A0A182RZ85). Using SWISS-MODEL, the template for *An*. *coluzzi* was identified as *An*. *gambiae* (PDB: 5YDH) with GMQE score of 0.81, GSQE score of 0.79, and 100% sequence identity with amino acid coverage from 162 to 699. The final QMEANDisCo Global was 0.93 ± 0.05. On the other hand, the best template identified for *An*. *funestus* was *An*. *gambiae* (PDB: 5YDI) with GMQE and GSQE scores of 0.80 and 0.74, respectively. This obtained 98.01% amino acid sequence identity with coverage from 160 to 696 and QMEANDisCo Global was 0.92 ± 0.05. The Ramachandran plots for *An*. *coluzzi* and *An*. *funestus* models obtained from the Ramachandran plot server [79] are reported in S2 and S3 Figs, respectively. Further, the local quality estimate results of the models and comparisons to non-redundant experimental crystal structures (obtained from SWISS-MODEL) are displayed in S4 and S5 Figs for *An*. *coluzzi* and *An*. *funestus*, respectively; followed by their validity verifications through Verify3D (S6 and S7 Figs, respectively). As a result, a comparison of the binding profile differences was performed using three new AChE models for *An*. *arabiensis*, *An*. *coluzzi*, and *An*. *funestus*, as well as two PDB sourced *An*. *gambiae* AChE targets; wild-type (PDB: 5YDI) [49] and resistant (PDB: 6ARY) through target site mutation (G280S) [78].

**Fig 9:**
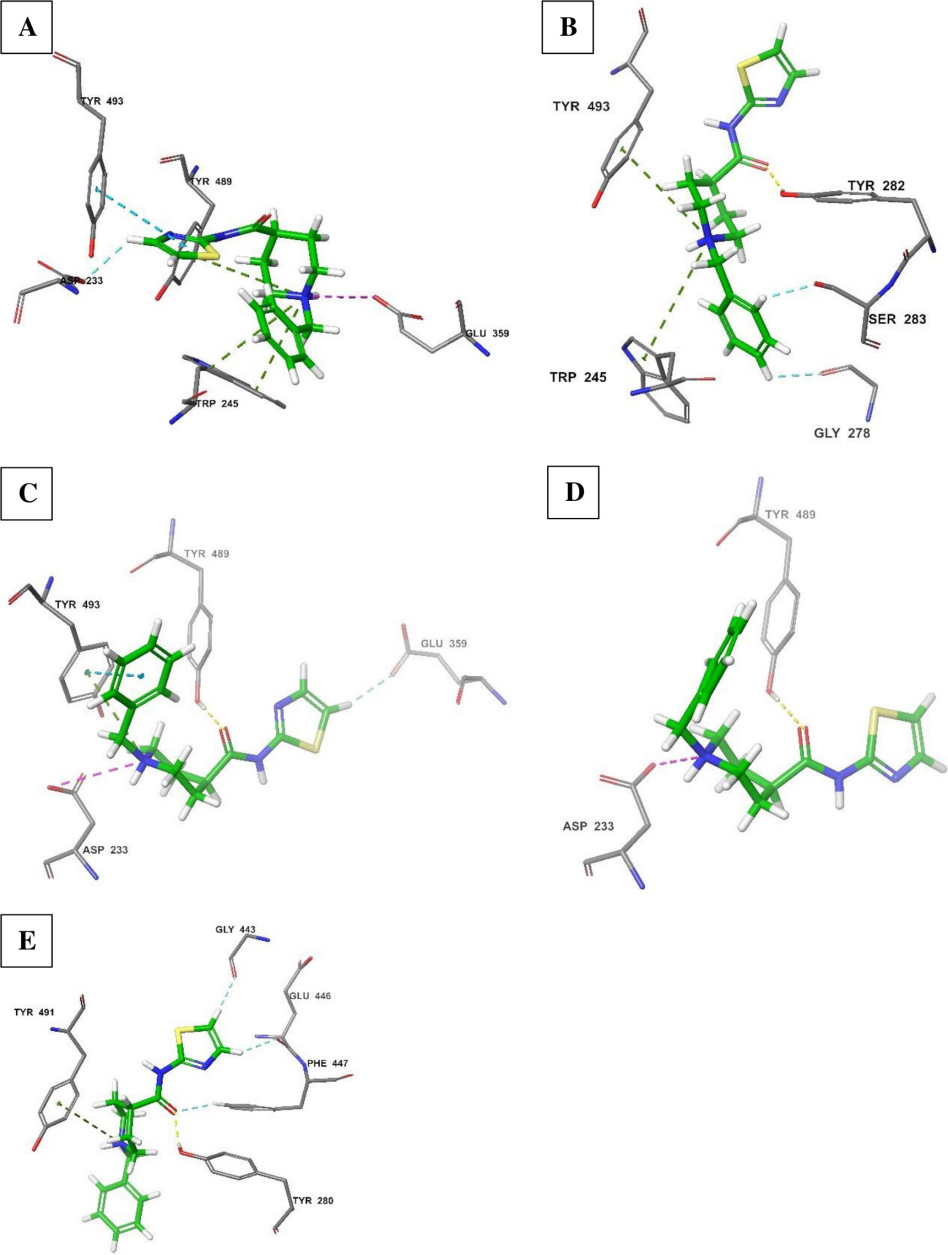
Comparison of the molecular interactions of derivative **2** with the AChE catalytic sites of *An*. *arabiensis* model (A; score: -7.5), wild-type *An*. *gambiae* (B; score: -9.2), target site mutated *An*. *gambiae* (C; score: -7.8), *An*. *coluzzii* model (D; score: -9.0) and *An*. *funestus* model (E; score: -9.1).

The observed stabilization of thiazole ring by Tyr^493^ in the *An*. *arabiensis* model (Fig 9A) could not be attained in the wild-type *An*. *gambiae* (Fig 9B). Similarly, the pi-cation stabilization by Tyr^489^ and Glu^359^ were also lost in the wild-type *An*. *gambiae* site. Instead, the interaction with the wild-type *An*. *gambiae* AChE showed aromatic hydrogen bonding between the *N*-benzylpiperidine ring and amino acid residues Gly^278^ and Ser^283^. The *N*-benzylpiperidine nitrogen atom was then involved in pi-cation interaction with aromatic amino acids, Trp^245^ and Tyr^493^, while Tyr^282^ established the hydrogen interaction with an oxygen group of the amide linker (Fig 9B). The generated crystal pose at this site was clearly different from that obtained with the *An*. *arabiensis* model. At the mutated target site of *An*. *gambiae* target (Fig 9C), the orientation of derivative **2** was clearly the opposite of that generated in *An*. *arabiensis* model and this displayed comparatively fewer intermolecular interactions. At this target, Glu^359^ was involved in hydrogen bonding with the thiazole group and the *N*-benzylpiperidine moiety was stabilized by Tyr^493^ and Asp^233^, in direct contrast to that observed with the *An*. *arabiensis* model where these Tyr^493^ and Asp^233^ stabilized the thiazole group. The Tyr^489^ held the molecule in space by establishing a hydrogen bond with the amide linker.

Least interactions were obtained against *An*. *coluzzii* target (Fig 9D). While the conformation of derivative **2** was similar to that obtained against mutant *An*. *gambiae* AChE (Fig 9C), where it only retained two interactions composed of Tyr^489^ H-bond and Asp^233^ pi-cation bond. Therefore, at both *An*. *gambiae* and *An*. *coluzzii* targets, derivative **2** assumed the orientation that was directly opposing that at *An*. *arabiensis* AChE catalytic site. Finally, at the *An*. *funestus* AChE site (Fig 9E), derivative **2** regenerated the crystal pose similar to that obtained at wild-type *An*. *gambiae* target (Fig 9B) with similar binding scores (-9.1 and -9.2 kcal/mol, respectively). In a similar fashion to wild-type *An*. *gambiae* AChE and different from *An*. *arabiensis* target, the pi-cation interaction with a tyrosine residue, Tyr^491^, was established and supplemented by the linker H-bond with Tyr^280^ and Phe^447^. At this target however, there was no further stabilization of the *N*-benzylpiperidine portion, but more H-bonds with the thiazole group from Gly^443^ and Glu^446^ residues (Fig 9E).

In general, derivative **2** generated a unique crystal pose against *An*. *arabiensis* model which may explain biological activity exclusively against this *Anopheles* species. On the other hand, this compound assumed a comparable pose between two targets, *An*. *funestus* wild-type and *An*. *gambiae* AChEs and established another conformation that was characteristic of *An*. *coluzzii* and mutant *An*. *gambiae* AChE.

As expected, this study showed great similarity between the AChEs from electric eel and human, in support of previous literature [4, 76, 77]. Interestingly, there were clear differences in ligand binding between either the electric eel or human AChE and *Anopheles* AChE, these differences potentially arising as a result of molecular differences around the catalytic site. In line with previous reports [75], the conserved Cys^447^ at the entrance to the catalytic site and Asp^602^ at the base of the active site were identified in this study. This molecular difference may bring about differences in ligand orientation, as well as interactions it establishes with surrounding amino acid residues. A smaller cysteine residue at the catalytic site entrance may create more volume available for a ligand to assume a different orientation as seen with a control, donepezil, and derivative **2**.

## 5. Conclusions

The current study identified a lead compound, 1-benzyl-*N*-(thiazol-2-yl) piperidine-4-carboxamide **2**, as an AChE-based larvicidal agent against *An*. *arabiensis* KWAG. Derivative **2** displayed more than 9-fold higher potency towards *An*. *arabiensis* AChE over that of electric eel AChE. Through molecular docking, this study has highlighted the close similarity between electric eel and human AChE, as well as their marked difference from *Anopheles* AChE. The differences in the binding of derivative **2** with electric eel and *An*. *arabiensis* AChE, respectively, were investigated at a molecular level through molecular modelling. Homology modelling was employed to generate a 3D AChE structure of *An*. *arabiensis* after which it was used for comparative binding assessments. The conformation of the molecule and interactions with the protein amino acid residues was noticeably different between the two targets. This informed our study to assess the molecular differences between the AChE binding sites from electric eel, human and *Anopheles*. A critical distinction observed in *Anopheles* as opposed to the two mammal proteins, is a smaller cysteine residue in place of bulky phenylalanine groups at the entrance to the catalytic site and a smaller aspartic acid replacing larger tyrosine residues at the base of the catalytic site, in correlation with previous X-ray diffraction studies [75]. The influence of these amino acid residues on ligand binding and crystal pose generation needs to be further investigated.

Further, the selectivity of derivative **2** to *An*. *arabiensis* amongst other *Anopheles* species was evaluated by comparing binding profiles between the *An*. *arabiensis* model and *An*. *gambiae* AChE targets (wild-type and resistant (mutant)) from the PDB repository, as well as those of *An*. *coluzzii* and *An*. *funestus* generated through homology modelling. The molecule was better stabilized by a mixture of hydrogen bonds and pi-pi stacking in the *An*. *arabiensis* model than in the corresponding *Anopheles* targets. In conclusion, this study suggests that 1-benzyl-*N*-(thiazol-2-yl) piperidine-4-carboxamide presents as a lead compound for the design and synthesis of novel insecticides against the African malaria vector, *An*. *arabiensis*. Further derivatizations of this molecule may generate molecules with high potency against a wide range of *Anopheles* species and increased selectivity to *Anopheles* over mammal AChE.

## Supporting information

S1 Fig The *An*. *arabiensis* AChE inhibitory activity of 2. (TIF)

S2 Fig Ramachandran plot for *An*. *coluzzii* model (98.90% amino acid residues in the highly preferred region (green crosses); 1.10% in the allowed region (orange triangles) and 0.00% in the questionable region (red circles)). (TIF)

S3 Fig Ramachandran plot for *An*. *funestus* model (98.34% amino acid residues in the highly preferred region (green crosses); 1.66% in the allowed region (orange triangles) and 0.00% in the questionable region (red circles)). (TIF)

S4 Fig a. Local quality estimate of the An. coluzzii model. b. Local quality estimate of the An. funestus model.
(ZIP)

S5 Fig a. Comparison of An. coluzzii model to the non-redundant experimental crystal structures. b. Comparison of An. funestus model to the non-redundant experimental crystal structures.
(ZIP)

S6 Fig *An*. *coluzzii* model validity verification through Verify3D (98.98% of the amino acid residues obtained a score of ≥0.2 in the 3D/1D profiling). (TIF)

S7 Fig *An*. *funestus* model validity verification through Verify3D (98.88% of the amino acid residues obtained a score of ≥0.2 in the 3D/1D profiling). (TIF)
