# Supplementary figures and images for: The in silico and in vitro analysis of donepezil derivatives for Anopheles acetylcholinesterase inhibition
Source: PLoS One. 2022 Nov 9;17(11):e0277363. doi: 10.1371/journal.pone.0277363 (PMC9645637; doi:10.1371/journal.pone.0277363)

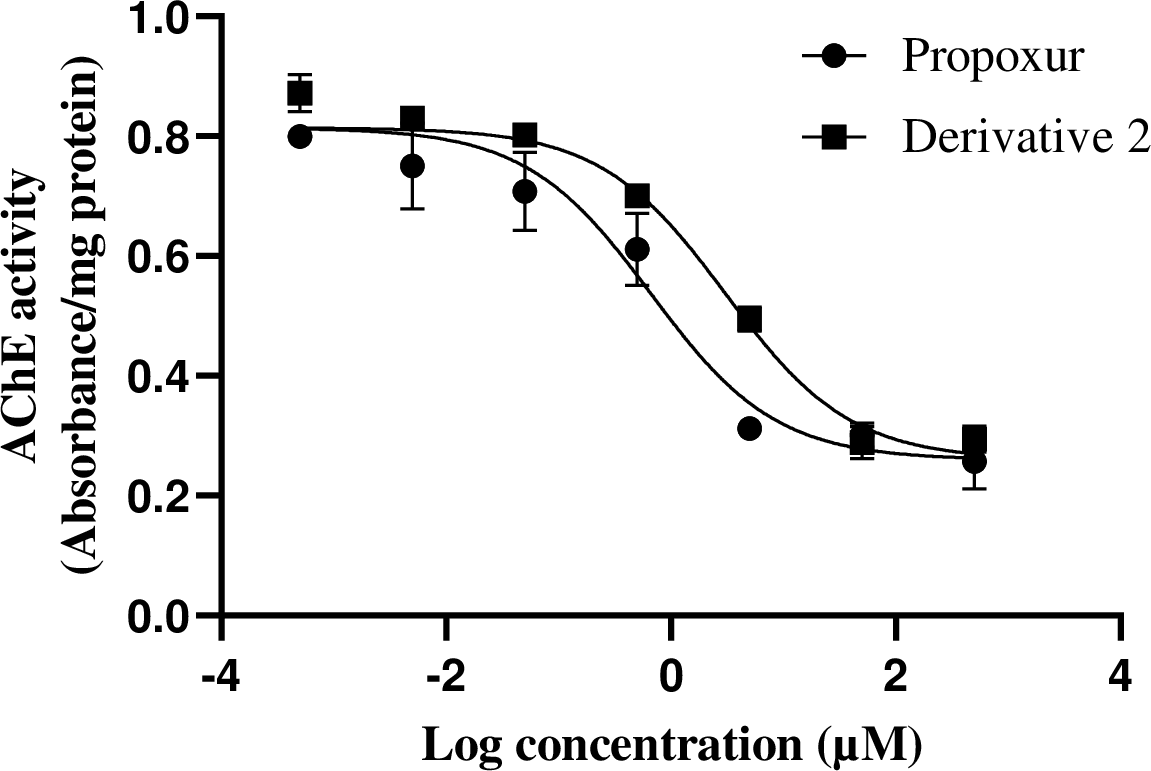

Supplement: S1 Fig — (TIF) [file pone.0277363.s001.tif]

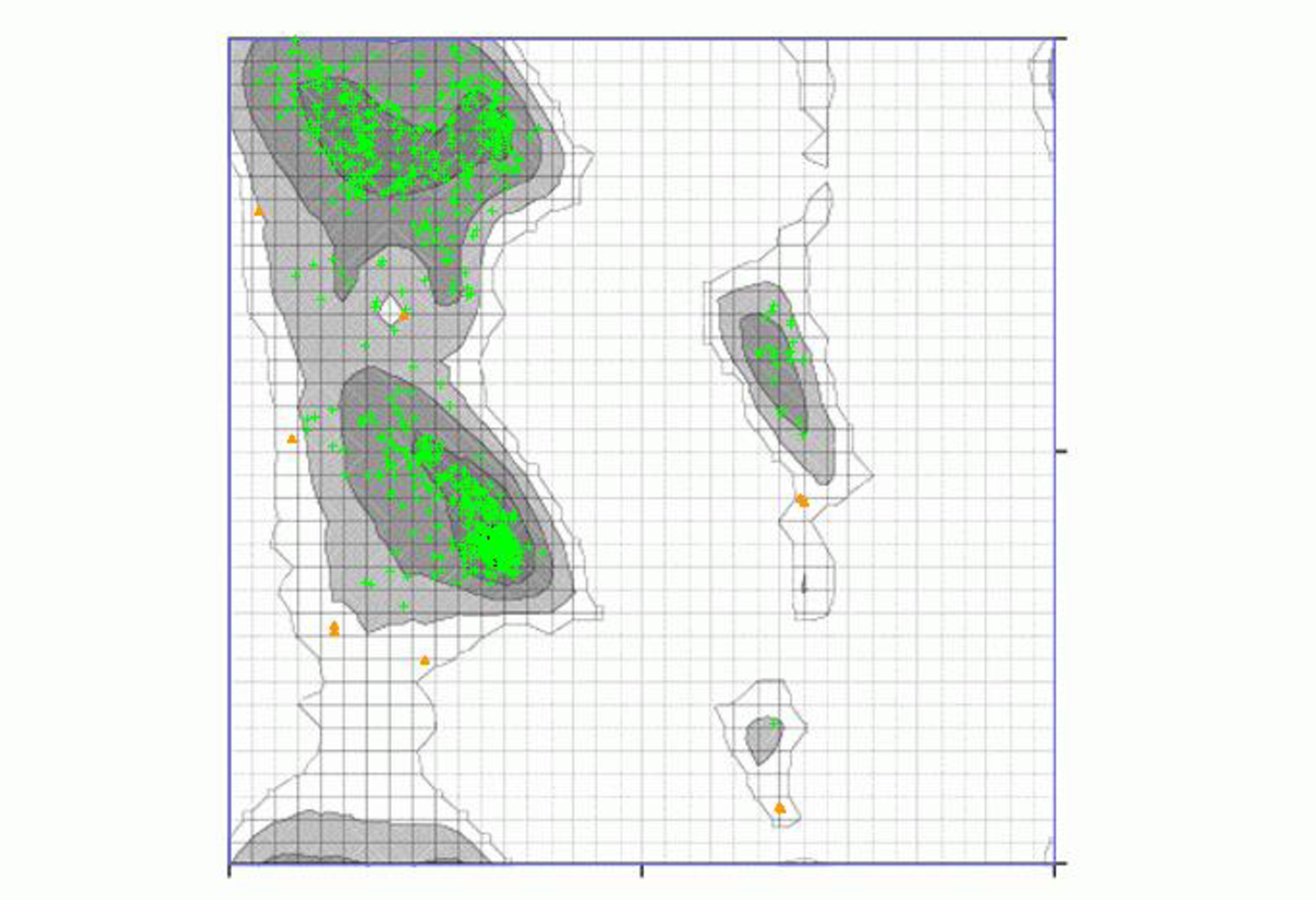

Supplement: S2 Fig — (TIF) [file pone.0277363.s002.tif]

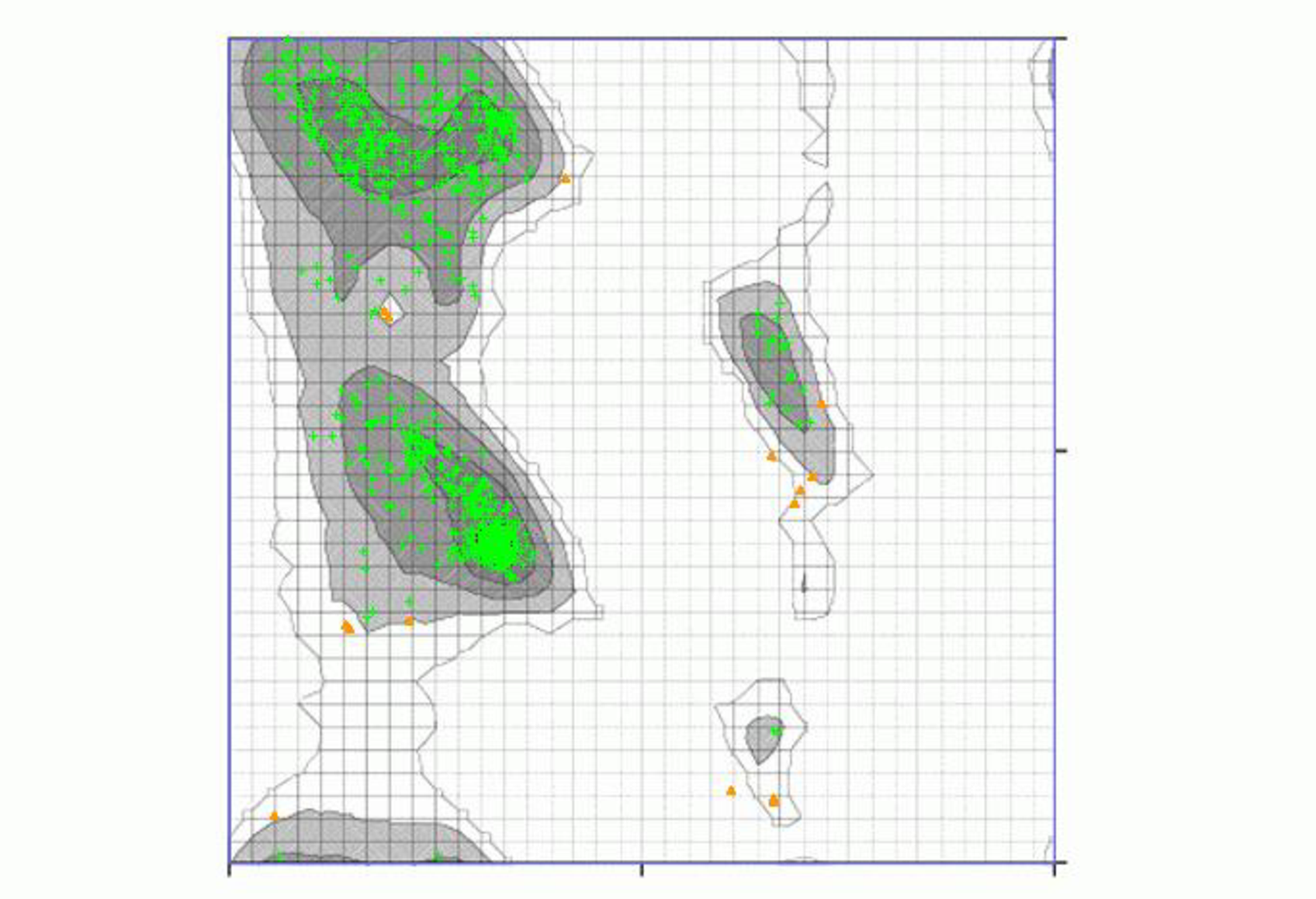

Supplement: S3 Fig — (TIF) [file pone.0277363.s003.tif]

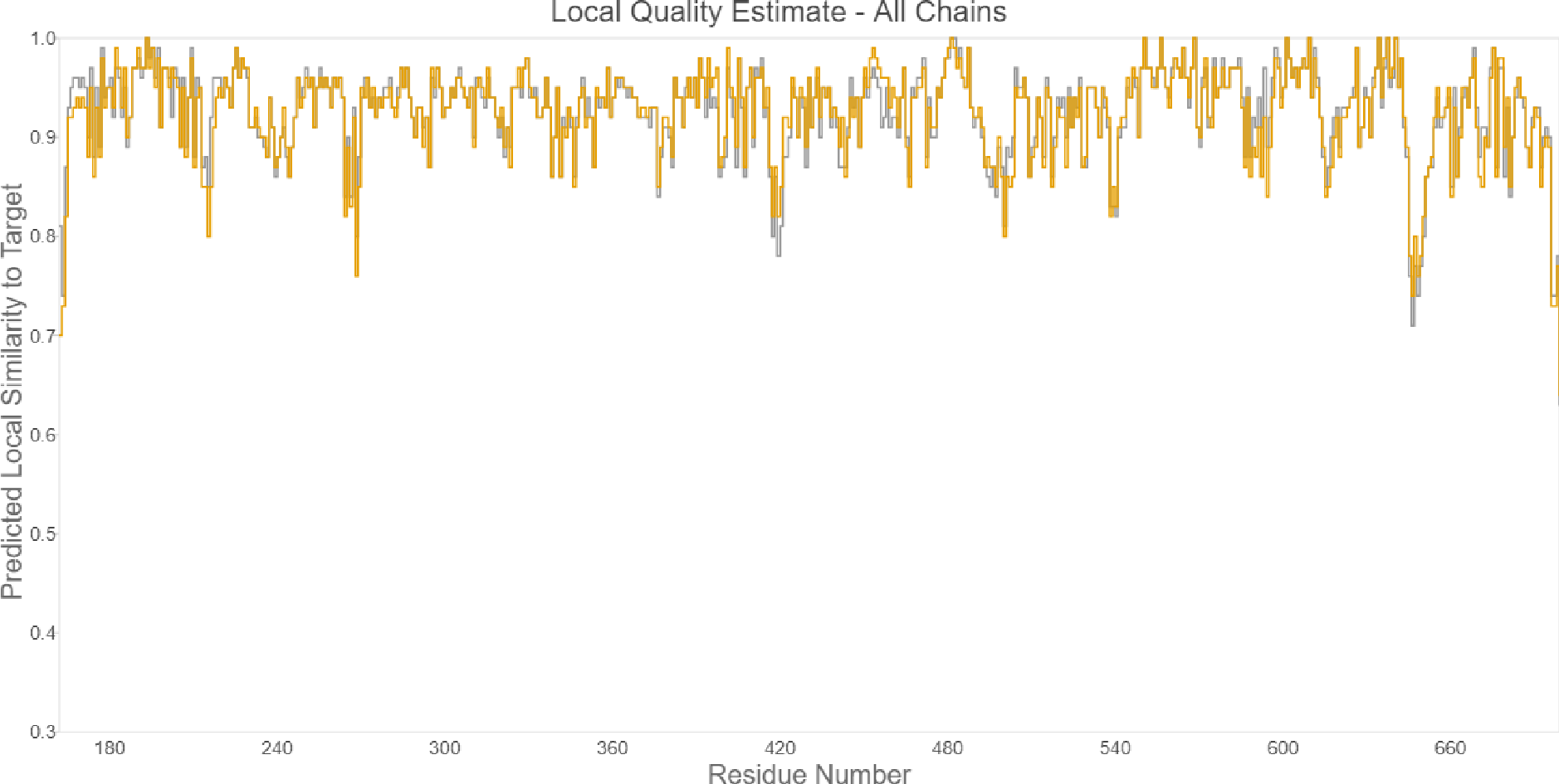

Supplement: S4 Fig — a. Local quality estimate of the An. coluzzii model. b. Local quality estimate of the An. funestus model. (ZIP) [file pone.0277363.s004.zip › S4a_Fig.tif]

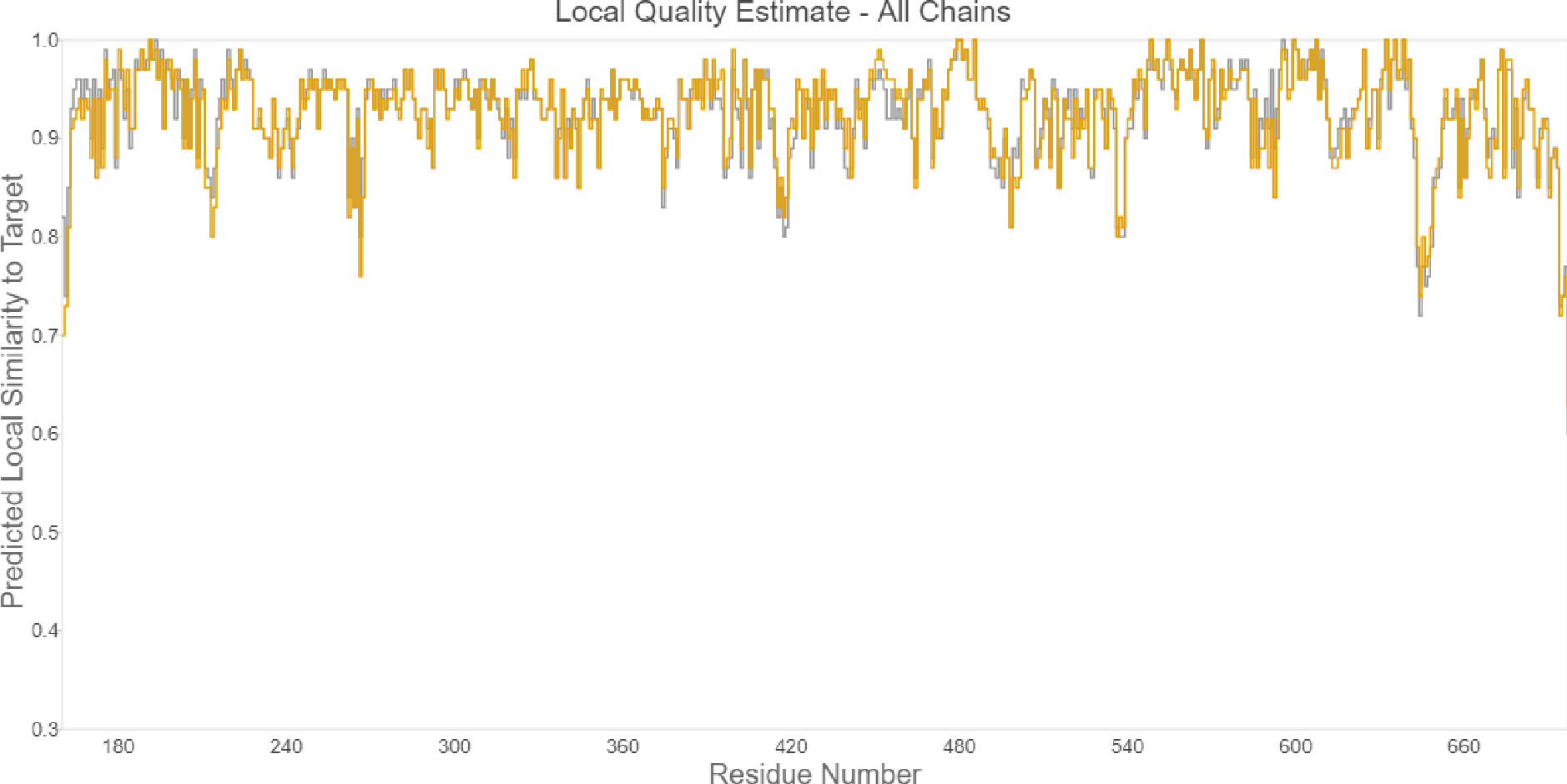

Supplement: S4 Fig — a. Local quality estimate of the An. coluzzii model. b. Local quality estimate of the An. funestus model. (ZIP) [file pone.0277363.s004.zip › S4b_Fig.tif]

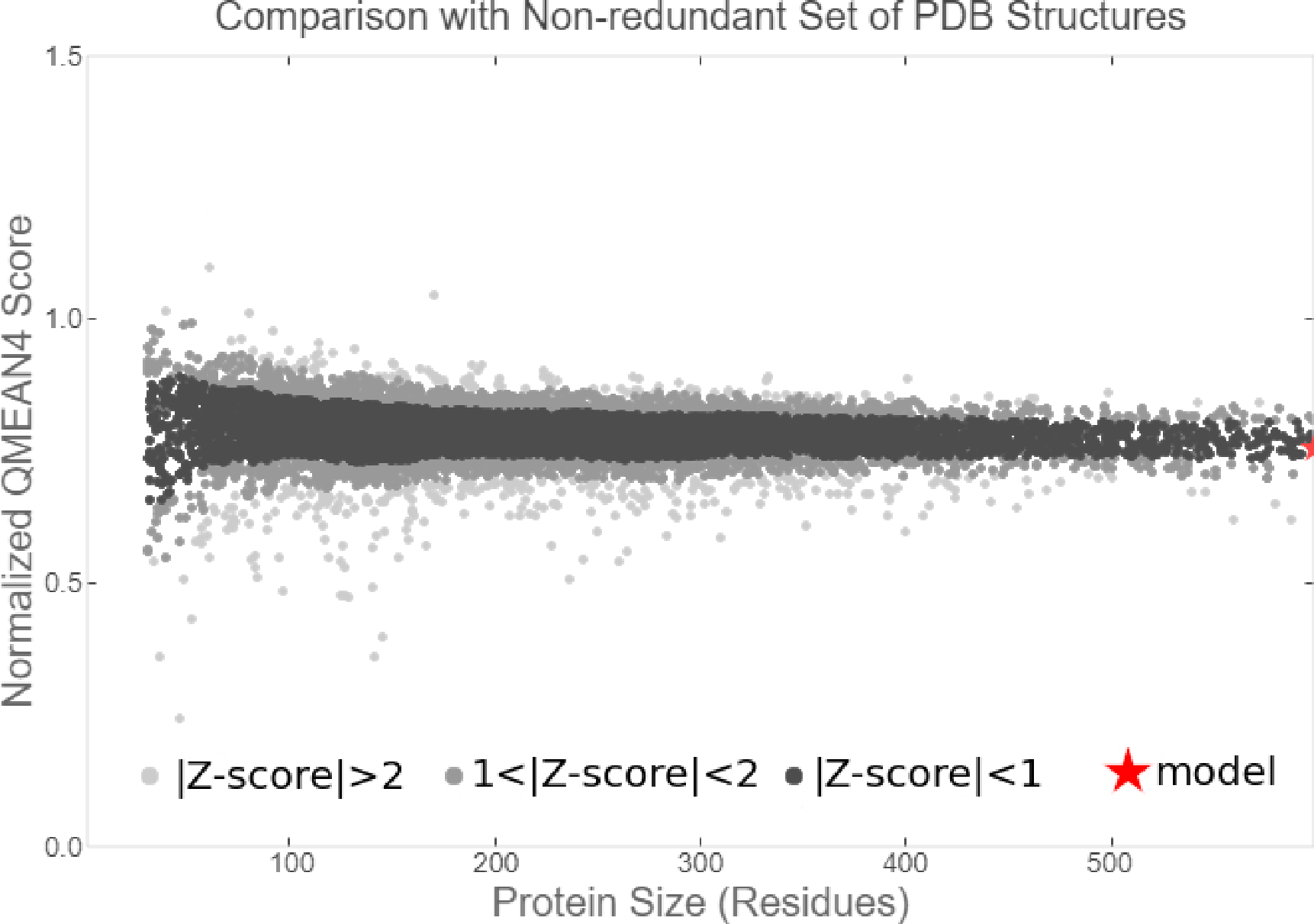

Supplement: S5 Fig — a. Comparison of An. coluzzii model to the non-redundant experimental crystal structures. b. Comparison of An. funestus model to the non-redundant experimental crystal structures. (ZIP) [file pone.0277363.s005.zip › S5b_Fig.tif]

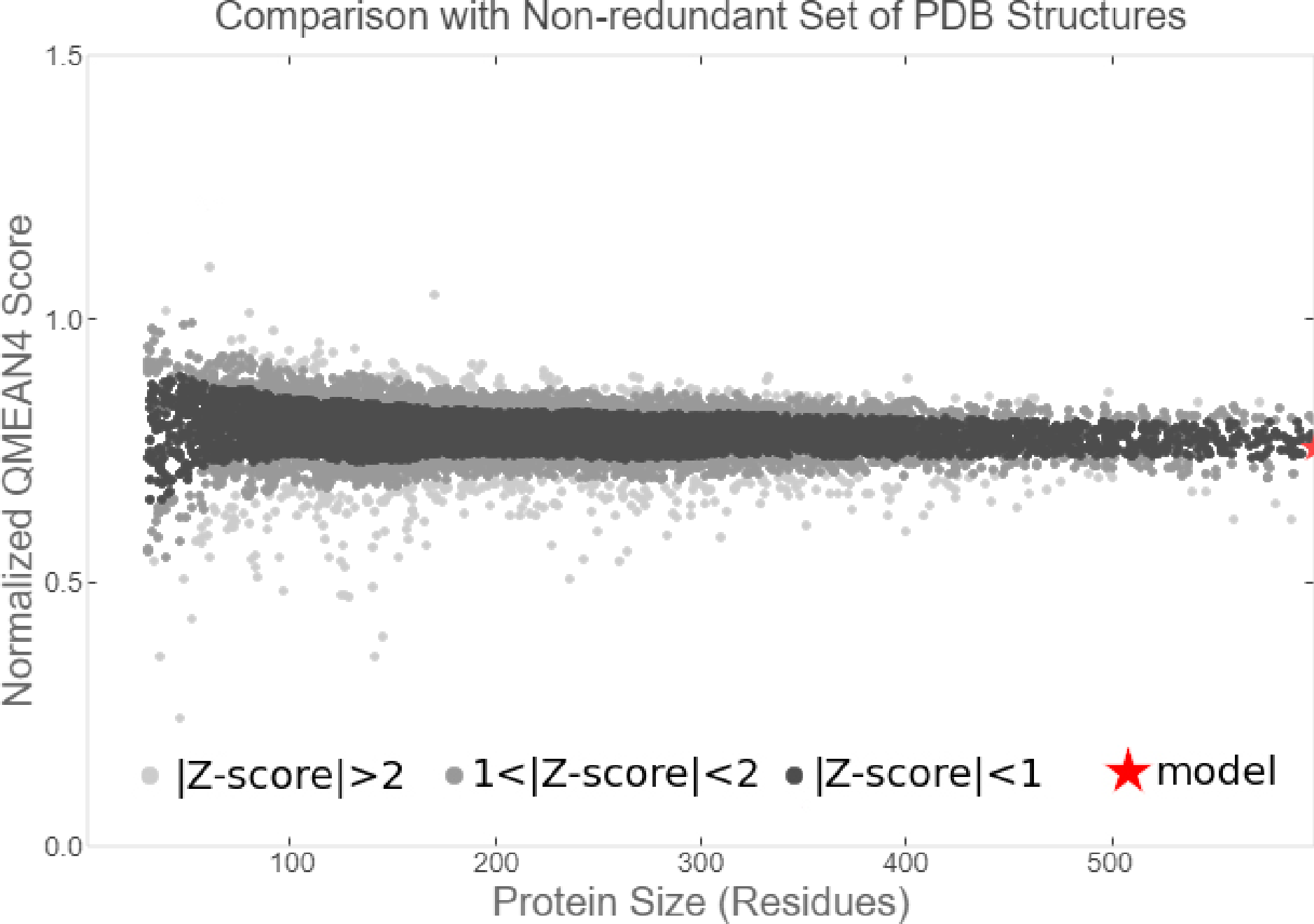

Supplement: S5 Fig — a. Comparison of An. coluzzii model to the non-redundant experimental crystal structures. b. Comparison of An. funestus model to the non-redundant experimental crystal structures. (ZIP) [file pone.0277363.s005.zip › S5a_Fig.tif]

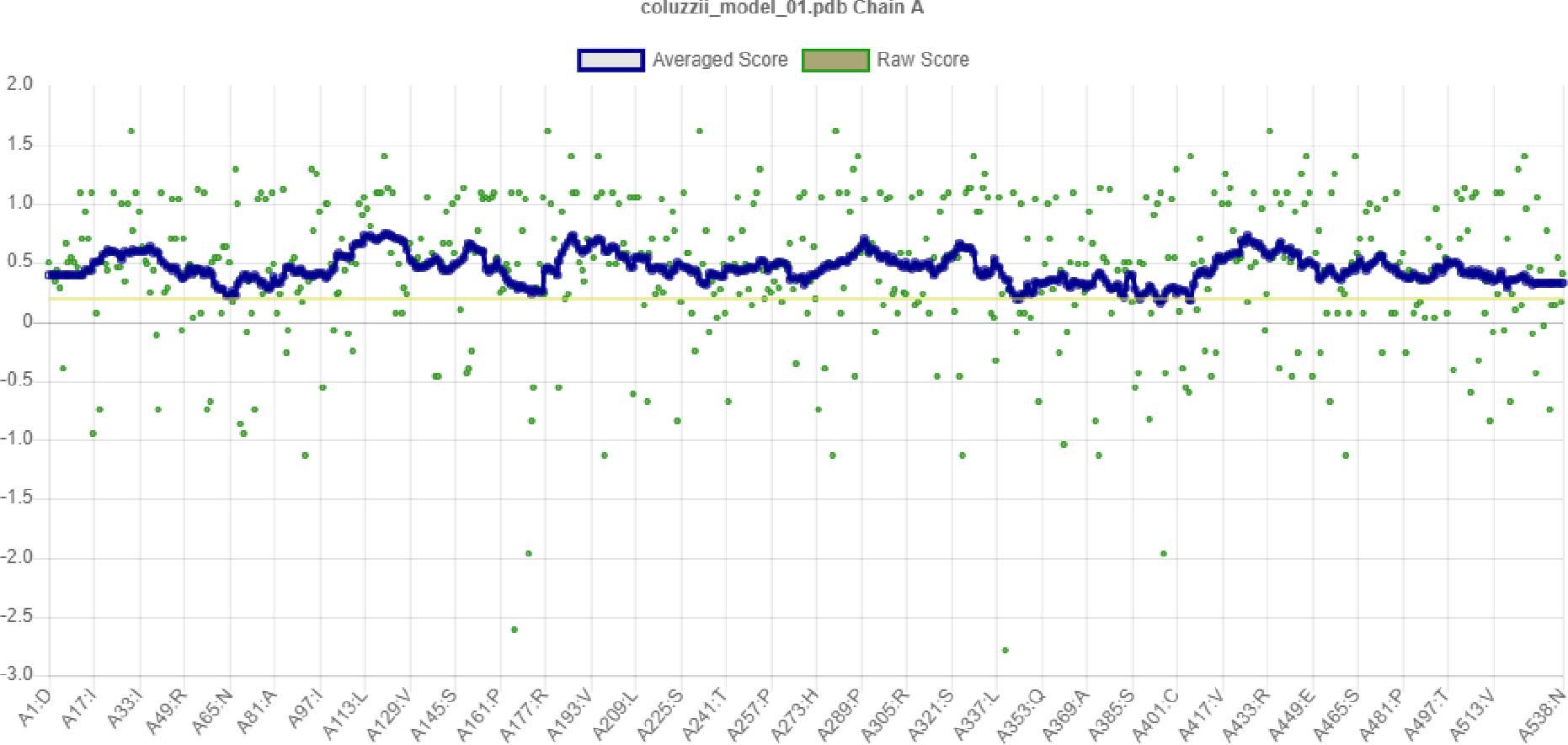

Supplement: S6 Fig — (TIF) [file pone.0277363.s006.tif]

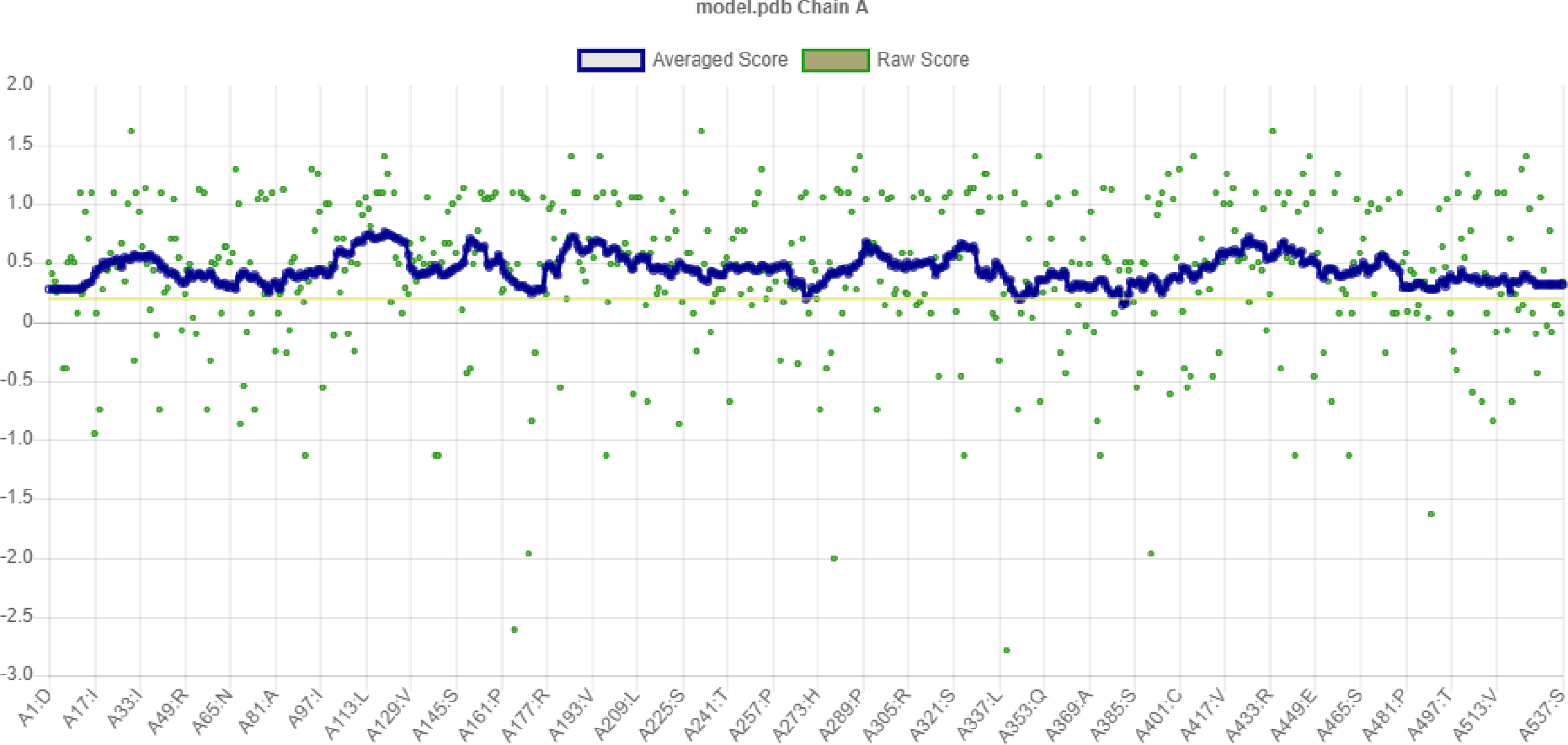

Supplement: S7 Fig — (TIF) [file pone.0277363.s007.tif]
